# Inhibition of Janus Kinase 1 synergizes docetaxel sensitivity in prostate cancer cells

**DOI:** 10.1111/jcmm.16684

**Published:** 2021-07-28

**Authors:** Geetha Nalairndran, Ivy Chung, Azad Hassan Abdul Razack, Felicia Fei‐Lei Chung, Ling‐Wei Hii, Wei‐Meng Lim, Chin King Looi, Chun‐Wai Mai, Chee‐Onn Leong

**Affiliations:** ^1^ Department of Pharmacology Faculty of Medicine University of Malaya Kuala Lumpur Malaysia; ^2^ University of Malaya Cancer Research Institute Faculty of Medicine University of Malaya Kuala Lumpur Malaysia; ^3^ Department of Surgery Faculty of Medicine University of Malaya Kuala Lumpur Malaysia; ^4^ Mechanisms of Carcinogenesis Section (MCA) Epigenetics Group (EGE) International Agency for Research on Cancer World Health Organization Lyon CEDEX 08 France; ^5^ Center for Cancer and Stem Cell Research Institute for Research Development and Innovation (IRDI) International Medical University Kuala Lumpur Malaysia; ^6^ School of Pharmacy International Medical University Kuala Lumpur Malaysia; ^7^ School of Postgraduate Studies International Medical University Kuala Lumpur Malaysia; ^8^ State Key Laboratory of Oncogenes and Related Genes Renji‐Med X Clinical Stem Cell Research Center Department of Urology Ren Ji Hospital School of Medicine Shanghai Jiao Tong University Shanghai China

**Keywords:** baricitinib, JAK1, prostate cancer, RNAi screen, ruxolitinib

## Abstract

Prostate cancer (PCa) is the second most common malignancy and is the fifth leading cause of cancer mortality among men globally. Docetaxel‐based therapy remains the first‐line treatment for metastatic castration‐resistant prostate cancer. However, dose‐limiting toxicity including neutropenia, myelosuppression and neurotoxicity is the major reason for docetaxel dose reductions and fewer cycles administered, despite a recent study showing a clear survival benefit with increased total number of docetaxel cycles in PCa patients. Although previous studies have attempted to improve the efficacy and reduce docetaxel toxicity through drug combination, no drug has yet demonstrated improved overall survival in clinical trial, highlighting the challenges of improving the activity of docetaxel monotherapy in PCa. Herein, we identified 15 lethality hits for which inhibition could enhance docetaxel sensitivity in PCa cells via a high‐throughput kinome‐wide loss‐of‐function screen. Further drug‐gene interactions analyses identified Janus kinase 1 (JAK1) as a viable druggable target with existing experimental inhibitors and FDA‐approved drugs. We demonstrated that depletion of endogenous JAK1 enhanced docetaxel‐induced apoptosis in PCa cells. Furthermore, inhibition of JAK1/2 by baricitinib and ruxolitinib synergizes docetaxel sensitivity in both androgen receptor (AR)–negative DU145 and PC3 cells, but not in the AR‐positive LNCaP cells. In contrast, no synergistic effects were observed in cells treated with JAK2‐specific inhibitor, fedratinib, suggesting that the synergistic effects are mainly mediated through JAK1 inhibition. In conclusion, the combination therapy with JAK1 inhibitors and docetaxel could be a useful therapeutic strategy in the treatment of prostate cancers.

## 1 INTRODUCTION

Prostate cancer (PCa) is the second most common cancer in men with an estimated worldwide incidence and mortality of 1.3 million new cases and 359,000 associated deaths in 2018, respectively.^1^ Despite aggressive screening and public health promotion, the global burden of PCa is anticipated to rise by 2030.^2^ Furthermore, despite dramatic shifts in treatment options over the last 15 years and recent advancements in targeted therapeutics, survival remains low in men presenting with metastatic disease, for whom median overall survival is reported to be 42.1 months (IQR: 22.7‐90.7 months).^3^

Docetaxel‐based therapy is the current first‐line chemotherapy for metastatic castration‐resistant prostate cancer (CRPC) with a response rate ranging from 17% to 33%.^4^, ^5^, ^6^ Dose‐limiting toxicity, including neutropenia, myelosuppression and neurotoxicity, is the major challenge that cause docetaxel dose reductions and eventually fewer cycles administered, despite recent study showing a clear association of survival benefit with docetaxel in PCa and total number of cycles administered.^6^ Several studies have attempted to improve the efficacy and reduce the toxicity of docetaxel through combination therapies, but have failed to improve the overall survival of PCa in clinical trials.^7^, ^8^, ^9^, ^10^, ^11^, ^12^, ^13^ Thus, the discovery of new druggable targets that could enhance docetaxel sensitivity in PCa is imperative.

Recent studies have demonstrated that ‘druggable’ pathways that regulate the survival of PCa cells can be identified through comprehensive loss‐of‐function RNA interference (RNAi) screens.^14^, ^15^, ^16^ Herein, we describe a systematic and comprehensive approach in employing a high‐throughput kinome‐wide shRNA screen coupled with in silico drug‐gene interaction analyses to uncover druggable targets that could enhance docetaxel sensitivity in PCa cells. We identified Janus kinase 1 (JAK1) as a druggable target that regulates docetaxel sensitivity. Depletion of endogenous JAK1 by shRNA or inhibition of JAK1 activities by JAK1 inhibitors synergized docetaxel sensitivity in AR‐negative DU145 and PC3 cells, but not in the AR‐positive LNCaP cells. Furthermore, we showed that JAK1 acts through activation of STAT3 in AR‐negative PCa cells. These results suggest that combination therapy with JAK1 inhibitor and docetaxel may be a useful therapeutic strategy in the treatment of PCa and warrants further investigation.

## 2 MATERIALS AND METHODS

### 2.1 Cell lines and cell cultures

DU145, PC3 and LNCaP human prostate cancer cell lines were acquired from American Type Culture Collection (ATCC) (Manassas, VA, USA) and maintained in RPMI 1640 (Sigma‐Aldrich, St Louis, MI, USA) with supplementation of 10% foetal bovine serum (FBS) (DNA Biotechnology, Science Park Road, Singapore), 100 IU/mL penicillin and 100 µg/mL streptomycin (Biowest, Nuaille, France). The RWPE‐1 normal prostate cell line was acquired from ATCC and grown in keratinocyte serum‐free medium consisting of 5 ng/mL of recombinant epidermal growth factor and 0.05 mg/mL of bovine pituitary extract (Invitrogen, Carlsbad, CA, USA). All cells were maintained in their logarithmic growth and kept in a humidified 37°C, 5% CO_2_ incubator.

### 2.2 Human kinome shRNA library screen

Briefly, screening was conducted using the MISSION LentiExpress^™^ Human Kinases shRNA Library (Sigma, St Louis, MO, USA) which consists of approximately 3200 lentiviral shRNA constructs targeting different regions of 501 human kinase genes. Firstly, the androgen receptor (AR)–negative DU145 cells were seeded at 2500 cells/well overnight in a white flat‐bottomed 384 well plate. Cells were transduced with 2500 lentivirus particles (multiplicity of infection, MOI = 1) in the presence of 7.5μg/ml polybrene (Sigma, St Louis, MO, USA) for 16 hours in 37°C, 5% CO_2_. After 18 hours of incubation, the medium containing the lentivirus particles was replaced with complete medium with the addition of a sublethal concentration of docetaxel (0.1 nM), and the cell viability was evaluated using the CellTiter‐Glo^®^ Luminescent Cell Viability Assay (Promega, Madison, WI, USA) at 72 hours post‐transduction. Controls include lentiviral particles carrying an empty vector (pLKO.1‐puro) and a non‐target shRNA (NS) to monitor transduction efficiency. All data were normalized against NS controls. The sensitivity index (SI) for each shRNA was calculated as described previously^17^, ^18^, ^19^, ^20^: SI = (Rc/Cc*Cd/Cc)‐(Rd/Cc), where Rc is the viability of cells following shRNA transduction (shRNA only), Rd is the viability of cells in following shRNA transduction and docetaxel treatment (shRNA + docetaxel), Cc is the average viability in cells transduced with NS (NS control), and Cd is the average viability in cells transduced with NS and docetaxel treatment (NS + docetaxel). Positive SI values indicate sensitizing effects, and negative value indicate antagonizing effects. Target genes were identified as hits when shRNA targeting a specific gene achieved a SI of more than 0.2.

### 2.3 Protein isolation and Western blot analysis

Protein lysates from the cells were extracted in an ice‐cold lysis buffer (1% NP‐40, 1 mM DTT, protease inhibitors and phosphatase inhibitor I and II cocktails in PBS as described previously).^14^, ^15^ A total protein of 50μg was loaded for immunoblotting. Primary antibodies against JAK1 and JAK2 were obtained from Cell Signaling Technology, MA, USA, and β‐actin was purchased from Santa Cruz Biotechnology, CA, USA.

### 2.4 Drug combination studies and analyses

Docetaxel and JAK inhibitors (baricitinib, ruxolitinib and fedratinib) were purchased from Selleckchem, Houston, TX, USA. For drug combination studies, cells were seeded overnight in 96‐well plates at a density of 5000 cells/well. Cells were treated with docetaxel and/or JAK inhibitors (baricitinib, ruxolitinib and fedratinib) alone or in combinations at dose‐response matrix format. Plates were then incubated at 37°C in a humidified 5% CO_2_ incubator for 72 hours. The plates were terminated by MTT cell proliferation assay at 72 hours after treatment. Calcusyn 2.1 software (Biosoft, Cambridge, UK) was used to generate combination index (CI) based on Chou‐Talalay method, in which CI <1, = 1 and >1 indicates synergism, additive and antagonism effect, respectively (Table S1).^21^, ^22^, ^23^, ^24^, ^25^ The dose‐response surface curves with levels of highest single agent (HSA) synergy were plotted by Combenefit software (Cancer Research UK Cambridge Institute).^21^, ^22^, ^26^

### 2.5 Lentiviral production and transduction

Lentiviral shRNA constructs targeting JAK1 were purchased from GE Healthcare Dharmacon Inc with target sequences shown in Table S2. High‐titre lentiviral stocks were generated by co‐transfecting the HEK‐293T cells with the psPAX packaging plasmid (Addgene plasmid 12260) and the pMD2.G envelope plasmid (Addgene plasmid 12259) using CalPhos Transfection Kits (Clontech, Mountain View, CA, USA) as described previously.^14^, ^15^, ^27^ Supernatants containing lentiviral stocks were supplemented with 7.5µg/mL polybrene (Sigma‐Aldrich, St Louis, MO, USA) for transduction. The stable pools of cells were generated through puromycin (Sigma‐Aldrich, St Louis, MO, USA) selection.

### 2.6 Detection of apoptosis by Annexin V flow cytometry

All floating and attached cells were stained for cell apoptosis assay using a PE Annexin V Apoptosis Detection Kit (BD Biosciences, San Jose, CA, USA) as described previously.^14^, ^15^ The samples were quantitated using a FACSCalibur flow cytometer and analysed by CellQuest Pro software (version 5.1.1; BD Biosciences, San Jose, CA, USA).

### 2.7 Transfection

Constitutively active EF.STAT3C. Ubc.GFP was a gift from Linzhao Cheng (Addgene plasmid # 24983; http://n2t.net/addgene:24983; RRID:Addgene_24983),^28^ and pcDNA3 Myr HA Akt1 was a gift from William Sellers (Addgene plasmid # 9008; http://n2t.net/addgene:9008; RRID:Addgene_9008).^29^ Plasmids were transfected into target cells using X‐tremeGENE HP DNA transfection reagent (Roche Diagnostic, Indianapolis, IN, USA), according to the manufacturer's protocol.

### 2.8 Statistical analysis

All results were presented as mean ± standard deviation (s.d.) from at least three independent experiments. Statistical significance was determined by Student's independent *t* test through SPSS (version 19.0 INC, Chicago, IL). A *P*‐value <0.05 was considered statistically significant.

## 3 RESULTS

### 3.1 DU145 and PC3 AR‐negative PCa cells are inherently resistant to docetaxel

**FIGURE 1:**
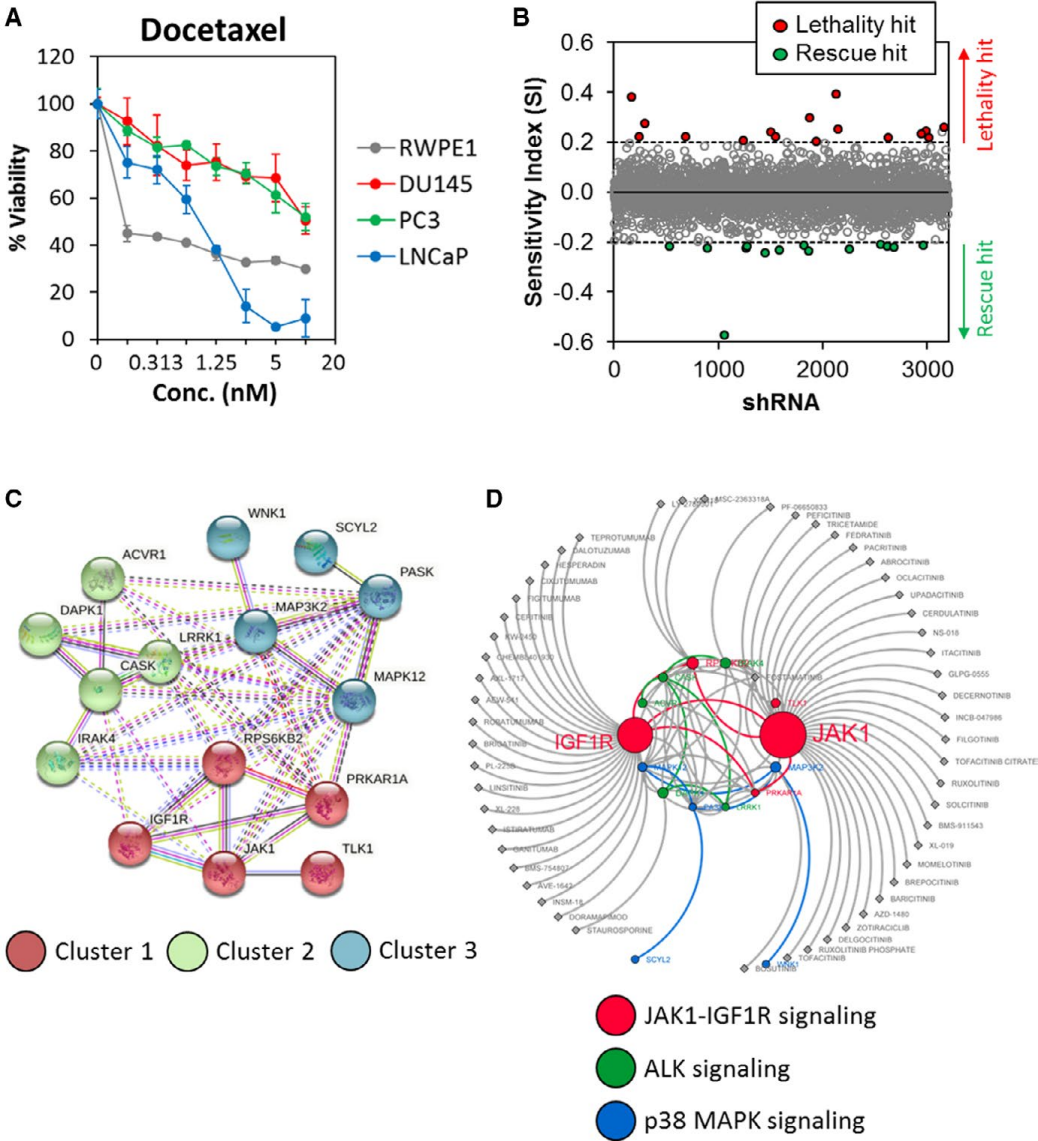
Kinome‐wide shRNA library screen identifies determinants of docetaxel sensitivity. (A) DU145 and PC3 AR‐negative PCa cells are inherently resistant to docetaxel. RWPE‐1, DU145, PC3 and LNCaP cells were treated with different concentrations of docetaxel for 72 hours and cell viability was determined by MTT assays. Points represent the mean ± SD of 3 independent experiments. (B) Kinase shRNA screen scatter plot. Sensitivity Index (SI) was plotted on the y‐axis against 3109 corresponding shRNAs on the *x*‐axis. The red dot represents lethality hit (ie genes when knock‐down enhances docetaxel sensitivity) and green dots represent rescue hit (ie genes when knock‐down confers docetaxel resistance). (C) Protein‐protein interaction network and cluster analysis of the 15 lethality hits using STRING. The identified clusters (by k‐means) are coloured in red (cluster 1), green (cluster 2) and blue (cluster 3). Nodes represent proteins. The solid and the dotted lines indicate connections within the same and different clusters, respectively. (D) Drug‐gene interaction network. Data mining of potential inhibitors interacting with the lethality hits were extracted from the DGIdb database. Node size represents the number of interactions

We first determined the sensitivity of a panel of PCa cells and normal prostate epithelial cancer cells against docetaxel. As shown in Figure 1A and Table S3, the AR‐positive LNCaP PCa cells and RWPE‐1 normal prostate epithelial cells were significantly more sensitive to docetaxel with an IC_50_ of <0.16 nM and 0.88 ± 0.12 nM, respectively, while the AR‐negative DU145 and PC3 cells were inherently resistant to docetaxel with IC_50_ of >10 nM.

### 3.2 Identification of synthetic lethality genes with docetaxel in PCa cells

Next, to identify kinases of which knock‐down enhanced the docetaxel sensitivity in PCa cells, we conducted a high‐throughput kinome‐wide shRNA screen in the AR‐negative DU145 prostate cancer cells in the presence or absence of a sublethal concentration of docetaxel. A sensitivity index (SI) was calculated for each shRNA to define the sensitizing (lethality hit; SI >0.2) or antagonistic (rescue hit; SI < −0.2) effects on docetaxel. A total of 15 lethality hits and 16 rescue hits were identified in the primary screen (Figure 1B; Tables 1 and 2).

**TABLE 1:** Lethality hits

Gene symbol	Gene name	SI
CASK	Calcium/calmodulin dependent serine protein kinase	0.393
ACVR1	Activin A receptor type 1	0.383
TLK1	Tousled like kinase 1	0.298
LRRK1	Leucine‐rich repeat kinase 1	0.274
IRAK4	Interleukin 1 receptor associated kinase 4	0.261
PRKAR1A	Protein kinase camp‐dependent type I regulatory subunit alpha	0.252
MAPK12	Mitogen‐activated protein kinase 12	0.244
JAK1	Janus kinase 1	0.242
IGF1R	Insulin‐like growth factor 1 receptor	0.234
RPS6KB2	Ribosomal protein S6 kinase B2	0.224
PASK	PAS domain containing serine/threonine kinase	0.223
WNK1	WNK lysine‐deficient protein kinase 1	0.220
SCYL2	SCY1‐like pseudokinase 2	0.218
DAPK1	Death‐associated protein kinase 1	0.207
MAP3K2	Mitogen‐activated protein kinase kinase kinase	0.203

**TABLE 2:** Rescue hits

Gene symbol	Gene name	SI
YES1	YES proto‐oncogene 1, Src family tyrosine kinase	−1.248
EPHA1	EPH receptor A1	−1.026
PHKA1	Phosphorylase kinase regulatory subunit alpha 1	−0.652
CAMKV	Cam kinase‐like vesicle associated	−0.572
MAPK4	Mitogen‐activated protein kinase 4	−0.243
PHKA2	Phosphorylase kinase regulatory subunit alpha 2	−0.237
ABL1	ABL proto‐oncogene 1, non‐receptor tyrosine kinase	−0.231
ATR	ATR serine/threonine kinase	−0.225
TGFBR2	Transforming growth factor beta receptor 2	−0.223
TWF2	Twinfilin actin binding protein 2	−0.219
IKBKB	Inhibitor of kappa light polypeptide gene enhancer in B cells, kinase beta	−0.218
ALK	Anaplastic lymphoma receptor tyrosine kinase	−0.216
EPHA10	EPH receptor A10	−0.215
NPR1	Natriuretic peptide receptor 1	−0.214
NEK11	NIMA‐related kinase 11	−0.213
ROCK2	Rho‐associated coiled‐coil containing protein kinase 2	−0.209

Cluster analysis was performed on the 15 lethality hits using K‐means clustering method in STRING (Search Tool for the Retrieval of Interacting Genes/Proteins) (http://string‐db.org/) to identify groups within them so as to prioritize genes for experimental validation. The analysis resulted in 3 clusters (referred to as clusters 1, 2 and 3) (Figure 1C). Cluster 1 has five members and was found to be enriched in genes involved in cell proliferation and chemoresistance through JAK1‐IGF1R signalling. Genes in clusters 2 and 3 were found to be related to epithelial tumorigenesis and metastasis mediated by ALK and p38 MAPK signalling.

Consistent with our screening results, inhibition of IGF1R,^30^, ^31^, ^32^, ^33^, ^34^, ^35^, ^36^ JAK1^37^, ^38^ and ACVR1 (also known as ALK2)^39^, ^40^, ^41^ have been shown to inhibit PCa cell growth and progression, particularly in hormone‐refractory PCa; while MAPK2 and IRAK4 have been implicated in tumorigenesis in other cancers including colorectal cancer,^42^, ^43^ breast cancer,^44^ pancreatic ductal adenocarcinoma,^45^ chronic lymphocytic leukaemia cells,^46^, ^47^ mutant MYD88 L265P diffuse large B cell lymphoma^48^, ^49^, ^50^, ^51^ and melanoma.^52^ Together, these candidates represent promising lethality targets to enhance docetaxel sensitivity in PCa cells.

### 3.3 JAK1 as a potential druggable and repurposing candidate to enhance docetaxel sensitivity in PCa cells

To identify novel druggable targets that could enhance docetaxel sensitivity in PCa, we queried the lethality hits against the Drug Gene Interaction Database (DGIdb) and identified JAK1 and IGF1R as druggable targets with existing FDA‐approved or experimental inhibitors (Figure 1D). We note that monoclonal antibodies against IGF1R have already shown poor efficacy and increased docetaxel toxicity in PCa clinical trials.^53^, ^54^ In contrast, JAK inhibitors were shown to possess good safety profiles in randomized controlled trials and their long‐term extension studies have been demonstrated in various immune‐mediated diseases such as rheumatoid arthritis and psoriatic arthritis.^55^, ^56^ In addition, recent studies in PCa model also demonstrated that JAK1/2 inhibitors suppress the immune escape of castration‐resistant prostate cancer CRPC) to natural killer (NK) cells,^57^ inhibit PCa metastasis ^37^ and inhibit progression of CRPC.^38^ Hence, JAK1 represents a promising druggable target for repurposing existing drugs to enhance docetaxel sensitivity in PCa.

### 3.4 Depletion of endogenous JAK1 enhances docetaxel sensitivity in PCa cells

**FIGURE 2:**
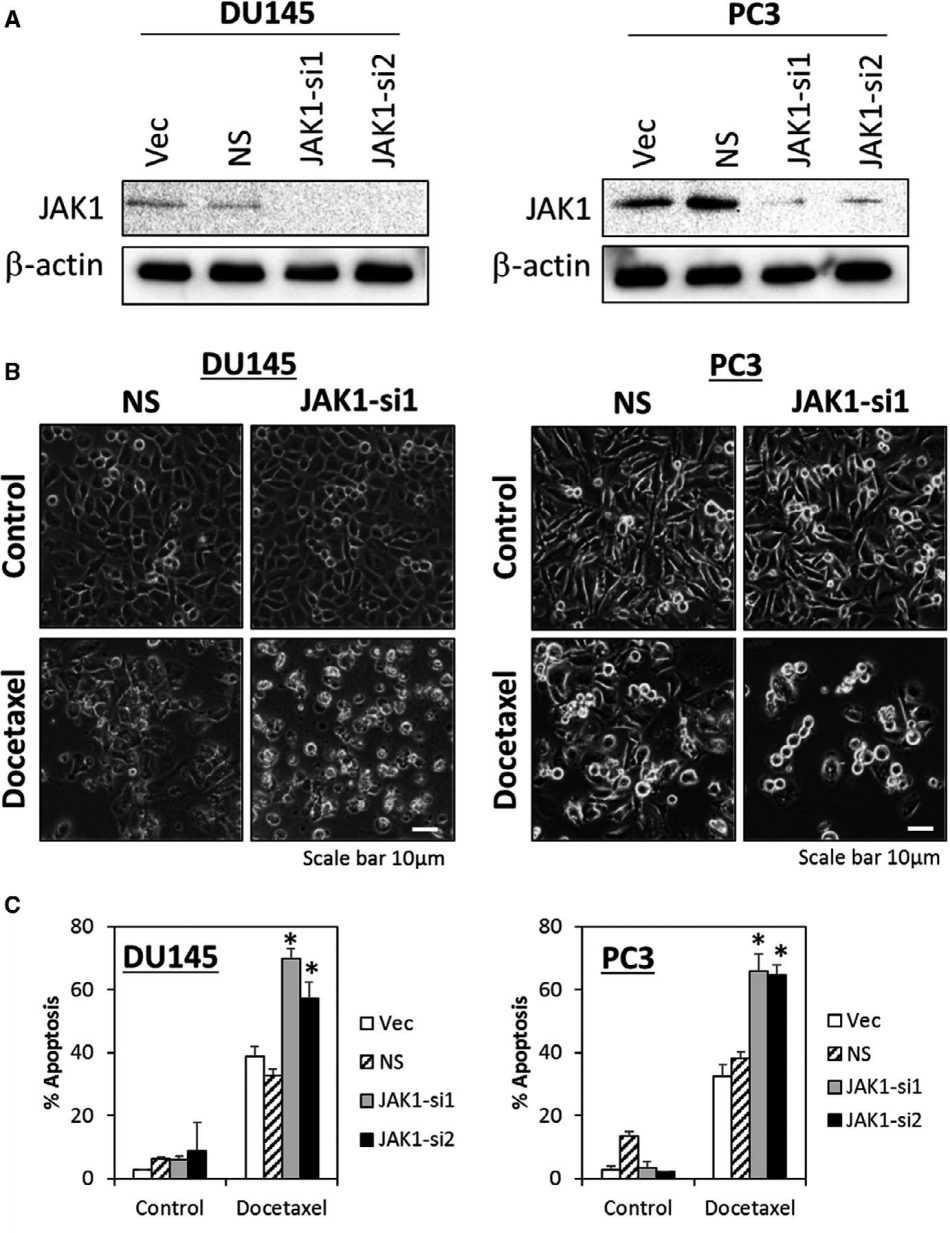
Depletion of endogenous JAK1 enhances docetaxel sensitivity in DU145 and PC3 cells. (A) Efficient knock‐down of endogenous JAK1 by two independent lentiviral shRNA constructs in DU145 and PC3 cells. Vector (Vec) and non‐target (NS) controls were included for accurate assessments of knock‐down efficiency; β‐actin was used as a loading control. (B) Morphological changes at 72 hours following 10 nM docetaxel treatment in DU145 and PC3 cells. Original magnification, ×100. (C) Increased apoptosis induced by docetaxel in JAK1‐depleted DU145 and PC3 cells. Cells were treated with 10 nM of docetaxel for 72 hours followed by quantitation of apoptosis using Annexin V/7‐AAD flow cytometry. Bars represent the means ± SD of three independent experiments. Asterisks (*) indicate statistical significance compared with docetaxel‐treated NS control cells (*P* < .01, Student's *t* test)

To directly validate the effects of JAK1 inhibition in enhancing docetaxel sensitivity in PCa cells, we generated stable pools of JAK1‐depleted isogenic cell lines in DU145 and PC3 cells. Efficient knock‐down of endogenous JAK1 by two independent shRNAs were demonstrated at the protein level for both shRNA constructs (JAK1‐si1 and JAK1‐si2) (Figure 2A). Significant increase in apoptosis was observed in the JAK1‐depleted cells following treatment with 10nM docetaxel as compared to treated vector or NS control cells, suggesting that depletion of endogenous JAK1 enhances docetaxel sensitivity in PCa cells (Figure 2B,C).

### 3.5 JAK1/2 inhibitors synergize docetaxel sensitivity in AR‐negative DU145 and PC3 PCa cells

**FIGURE 3:**
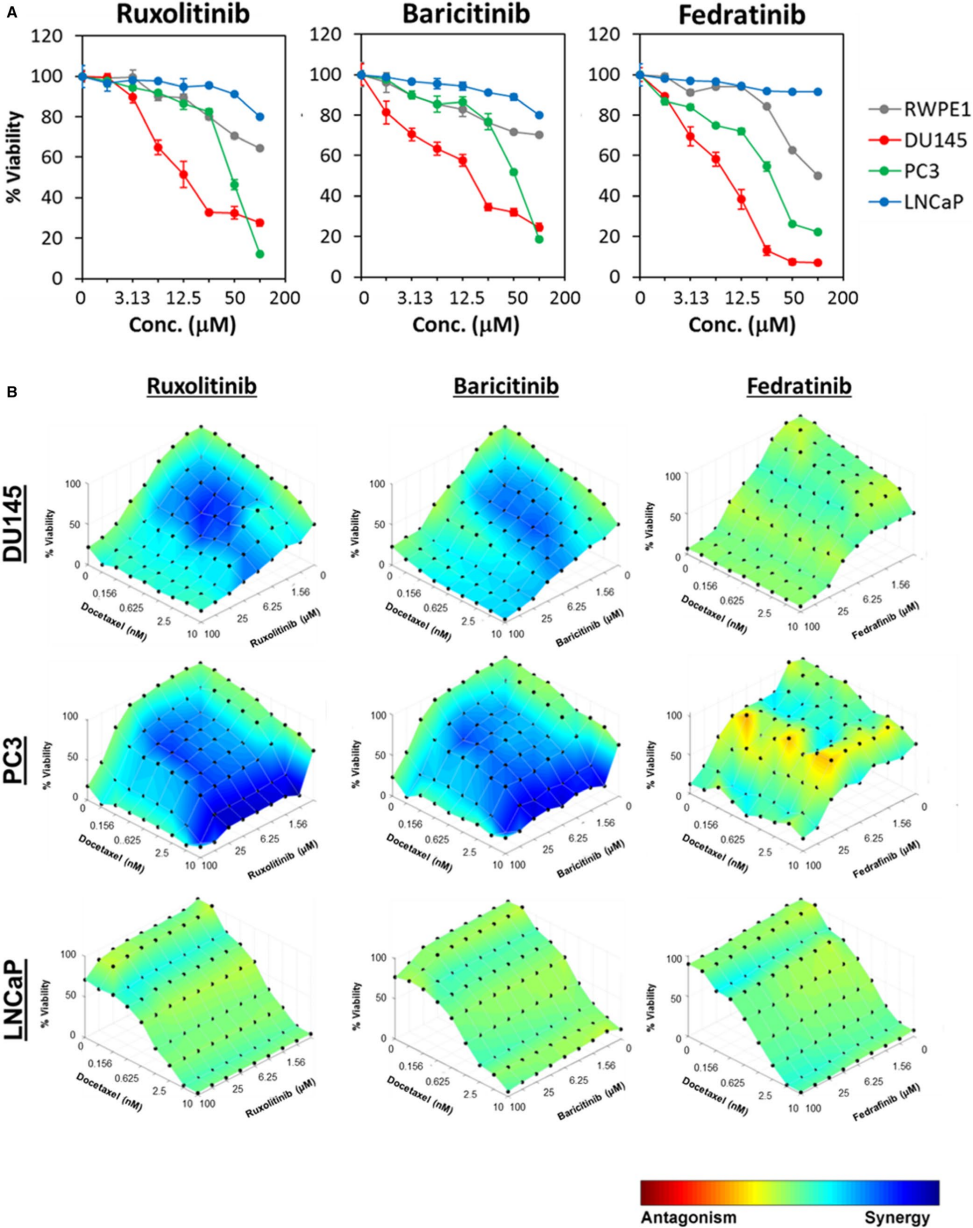
JAK1/2 inhibitors synergize docetaxel sensitivity in AR‐negative PCa cells. (A) Selective growth inhibitory effects of JAK1/2 inhibitors (ruxolitinib and baricitinib) and JAK2‐specific inhibitor (fedratinib) on PCa cells. DU145, PC3 and LNCaP prostate cancer cell lines, as well as the non‐transformed RWPE‐1 prostate cell line, were treated with increasing concentrations of JAK inhibitors. Cell viability was determined using the MTT cell viability assay 72 hours after JAK inhibitors treatment. Each data point represents the mean ± SD of at least three independent experiments. (B) Synergistic effects of ruxolitinib and baricitinib, in combination with docetaxel in AR‐negative DU145 and PC3, but not the AR‐positive LNCaP cells. Fedratinib exhibited mainly additive effects with docetaxel in DU145 and antagonistic effects in PC3. The efficacy of drug combinations was assessed by treating cells with serial dilutions of the inhibitors in an 8 × 8 combination matrix. Cell proliferation was evaluated in 96‐well plates, using MTT cell viability assay at 72 hours after treatment. Dose response surface curves and synergy for each combination was assessed using the HSA model (effect‐based approach), as implemented in Combenefit software. Level of synergism (blue) or antagonism (red) at each concentration is represented by colour scale bar. All experiments were conducted in at least three independent experiments

Next, we investigated the antitumor effects of selected JAK inhibitors (ruxolitinib, baricitinib and fedratinib). Of note, ruxolitinib and baricitinib are JAK1/2 inhibitors while fedratinib is a specific JAK2 inhibitor. Baricitinib, ruxolitinib and fedratinib induced selective antitumor effects against the AR‐negative DU145 and PC3 cells, while the AR‐positive LNCaP cells and the RWPE‐1 normal prostate epithelial cells were relatively less sensitive to the JAK1/2 inhibitors (Figure 3 and Table S3).

To test whether the JAK inhibitors could synergize docetaxel sensitivity in the PCa cells, we investigated the anti‐proliferative effects of baricitinib, fedratinib and ruxolitinib in combination with docetaxel in the DU145, PC3 and LNCaP cells. The anti‐proliferative effect of the combination treatment was evaluated via MTT assays. Analyses of drug interactions using the highest single agent model (HSA) and combination index indicated that the JAK1/2 inhibitors, ruxolitinib and baricitinib, synergized docetaxel sensitivity selectively in the AR‐negative DU145 and PC3 cells, but not in the AR‐positive LNCaP cells (Figure 3B; Tables 3, 4, 5). In contrast, the JAK2 inhibitor, fedratinib, exhibited mainly additive effects with docetaxel in DU145 and antagonistic effects in PC3 cells (Tables 3 and 4). Taken together, our findings demonstrate that inhibition of JAK1 synergized docetaxel sensitivity in AR‐negative PCa cells.

**TABLE 3:** Effects of JAK inhibitors combined with docetaxel in DU145 cells

Inhibitor	Inh:Doc Ratio	Combination Index (CI)			Mean CI	Interactions
		ED50	ED75	ED90		
Ruxolitinib (JAK1/2)	2500:1	0.284	0.436	0.706	0.475 ± 0.214	Synergism
	5000:1	0.271	0.411	0.646	0.443 ± 0.189	Synergism
	10 000:1	0.264	0.435	0.729	0.476 ± 0.235	Synergism
	20 000:1	0.308	0.481	0.760	0.516 ± 0.228	Synergism
	40 000:1	0.315	0.457	0.667	0.480 ± 0.177	Synergism
Baricitinib (JAK1/2)	2500:1	0.282	0.258	0.250	0.263 ± 0.017	Strong synergism
	5000:1	0.164	0.132	0.109	0.135 ± 0.028	Strong synergism
	10 000:1	0.347	0.296	0.255	0.299 ± 0.046	Strong synergism
	20 000:1	0.381	0.332	0.290	0.334 ± 0.045	Synergism
	40 000:1	0.516	0.386	0.290	0.397 ± 0.114	Synergism
Fedratinib (JAK2)	2500:1	0.932	0.911	0.947	0.930 ± 0.018	Nearly additive
	5000:1	0.957	0.894	0.862	0.904 ± 0.049	Nearly additive
	10 000:1	1.059	1.018	0.995	1.024 ± 0.032	Nearly additive
	20 000:1	0.916	0.954	1.003	0.958 ± 0.044	Nearly additive
	40 000:1	0.828	0.961	1.120	0.970 ± 0.146	Nearly additive

**TABLE 4:** Effects of JAK inhibitors combined with docetaxel in PC3 cells

Inhibitor	Inh:Doc ratio	Combination Index (CI)			Mean CI	Interactions
		ED50	ED75	ED90		
Ruxolitinib (JAK1/2)	2500:1	0.231	0.274	0.399	0.301 ± 0.087	Synergism
	5000:1	0.252	0.334	0.506	0.364 ± 0.130	Synergism
	10 000:1	0.201	0.307	0.509	0.339 ± 0.157	Synergism
	20 000:1	0.278	0.437	0.720	0.478 ± 0.224	Synergism
	40 000:1	0.374	0.601	0.990	0.655 ± 0.311	Synergism
Baricitinib (JAK1/2)	2500:1	0.321	0.341	0.380	0.347 ± 0.030	Synergism
	5000:1	0.366	0.427	0.521	0.438 ± 0.078	Synergism
	10 000:1	0.300	0.494	0.842	0.545 ± 0.274	Synergism
	20 000:1	0.331	0.533	0.876	0.580 ± 0.275	Synergism
	40 000:1	0.408	0.667	1.102	0.726 ± 0.351	Moderate synergism
Fedratinib (JAK2)	2500:1	8.780	> 10	> 10	> 10	Very strong antagonism
	5000:1	1.487	1.332	1.204	1.341 ± 0.142	Moderate antagonism
	10 000:1	1.379	1.128	0.928	1.145 ± 0.226	Slight antagonism
	20 000:1	1.433	1.479	1.531	1.481 ± 0.049	Antagonism
	40 000:1	1.144	0.992	0.861	0.999 ± 0.142	Nearly additive

**TABLE 5:** Effects of JAK inhibitors combined with docetaxel in LNCaP cells

Inhibitor	Inh:Doc Ratio	Combination Index (CI)			Mean CI	Interactions
		ED50	ED75	ED90		
Ruxolitinib (JAK1/2)	2500:1	1.209	1.058	0.928	1.065 ± 0.141	Nearly additive
	5000:1	1.039	0.980	0.925	0.981 ± 0.057	Nearly additive
	10 000:1	1.081	0.958	0.851	0.963 ± 0.115	Nearly additive
	20 000:1	1.041	0.919	0.813	0.925 ± 0.114	Nearly additive
	40 000:1	1.156	1.175	1.197	1.176 ± 0.021	Slight antagonism
Baricitinib (JAK1/2)	2500:1	1.104	1.030	0.961	1.032 ± 0.072	Nearly additive
	5000:1	1.039	0.974	0.914	0.976 ± 0.063	Nearly additive
	10 000:1	1.217	0.882	0.648	0.916 ± 0.286	Nearly additive
	20 000:1	1.172	0.865	0.646	0.894 ± 0.265	Slight synergism
	40 000:1	1.026	0.724	0.521	0.757 ± 0.254	Moderate synergism
Fedratinib (JAK2)	2500:1	1.121	0.993	0.861	0.992 ± 0.130	Nearly additive
	5000:1	1.124	0.987	0.957	1.022 ± 0.089	Nearly additive
	10 000:1	0.961	0.949	0.889	0.933 ± 0.038	Nearly additive
	20 000:1	0.989	0.929	0.845	0.921 ± 0.073	Nearly additive
	40 000:1	0.991	0.979	0.864	0.945 ± 0.071	Nearly additive

### 3.6 JAK1 inhibitors synergize docetaxel activity in PCa cells via inhibition of STAT3 signalling

JAK1 has been shown to play an important role in activating signal transducer and activator of transcription 3 (STAT3) and phosphatidyl inositol‐3‐kinase (PI3K/AKT) signalling.^58^, ^59^, ^60^ To test whether the synergistic effects of JAK1 inhibitors and docetaxel in PCa cells could be mediated through inhibition of STAT3 and/or PI3K/AKT signalling, we treated the DU145 and PC3 cells with an IC_50_ concentration of docetaxel (10nM) in the presence or absence of the JAK1 inhibitors (ruxolitinib and baricitinib) followed by evaluating the effects of the combinations on STAT3 and AKT phosphorylation.

**FIGURE 4:**
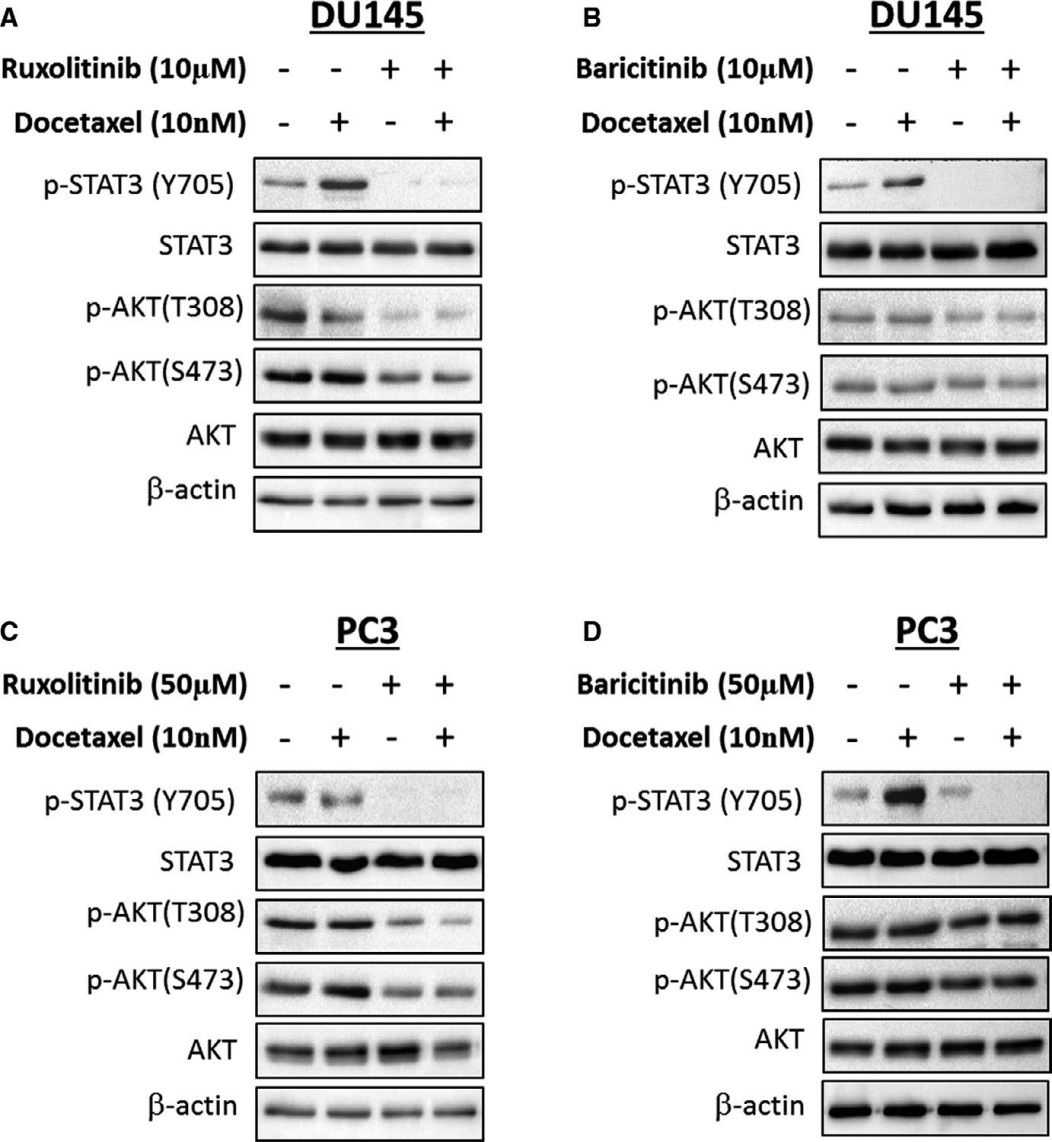
Effects of JAK1 inhibitors and/or docetaxel on STAT3 and PI3K/AKT signalling. (A and B) DU145 and (C and D) PC3 cells were treated with docetaxel in the presence or absence of JAK1 inhibitors (baricitinib and ruxolitinib) for 72 hours, and protein expression was analysed by immunoblotting

As shown in Figure 4A‐D, both ruxolitinib and baricitinib JAK1 inhibitors significantly reduced STAT3 phosphorylation in both DU145 and PC3 cells. In contrast, down‐regulation of phosphor‐AKT was only observed in cells treated with ruxolitinib (Figure 4A and C), but not baricitinib (Figure 4B and D).

**FIGURE 5:**
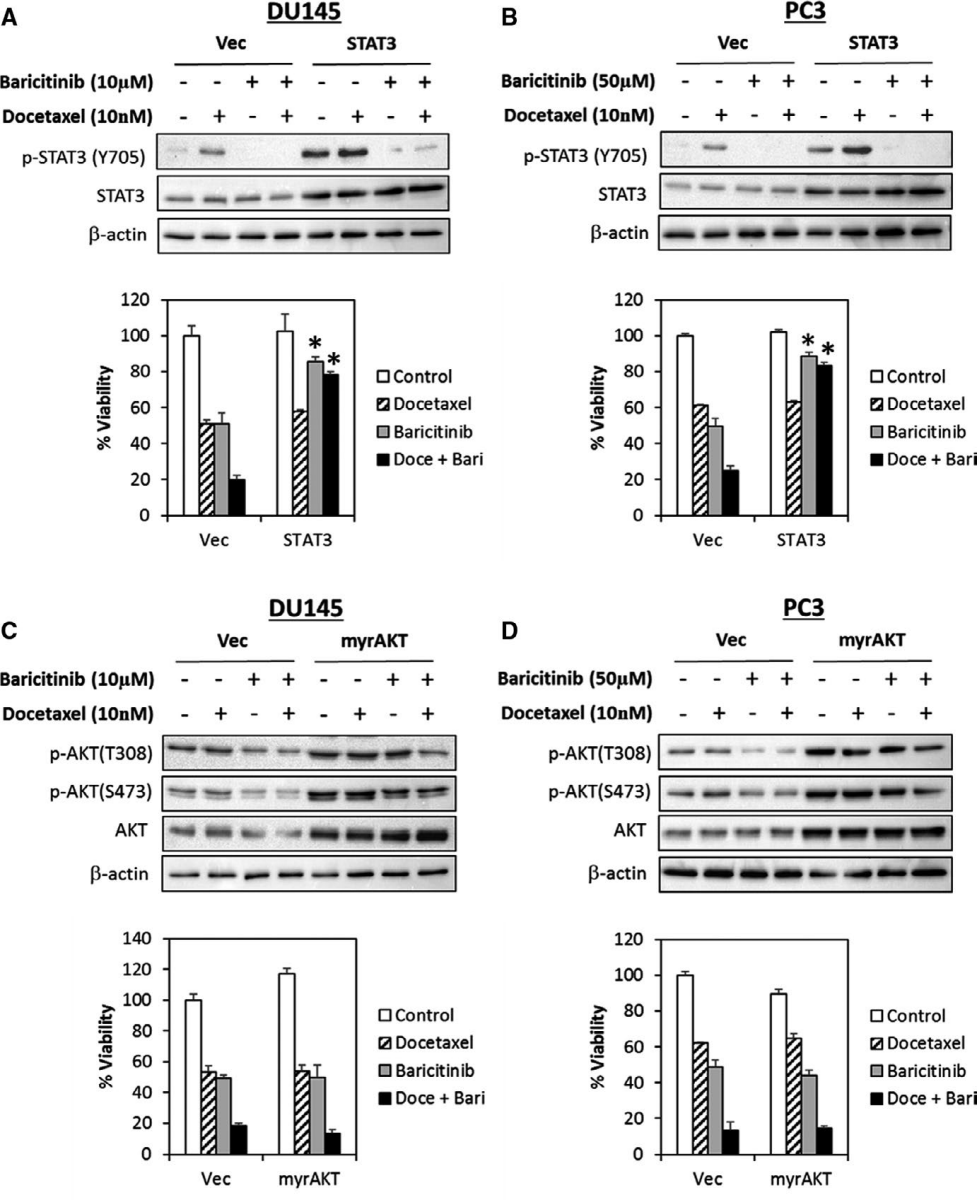
JAK1 inhibitors synergize docetaxel activity in PCa cells via inhibition of STAT3 signalling. (A and B) DU145 and PC3 cells were reverse‐transfected with a constitutively active STAT3 or (C and D) myristoylated AKT, followed by treatment of cells with docetaxel and/or baricitinib for 72 hours. Protein expression was analysed by immunoblotting. Cell viability was determined by the MTT assay. Bars represent the means ± SD of three independent experiments. Asterisks (*) indicate statistical significance compared with docetaxel‐ and or baricitinib‐treated vector control cells (*P* <.01, Student's *t* test)

Finally, to test whether the synergistic effects of JAK1 inhibitors and docetaxel in PCa cells is dependent on STAT3 or PI3K/AKT signalling, we ectopically expressed a constitutively active STAT3 or a myristoylated AKT in DU145 and PC3 cells followed by docetaxel treatment in the presence or absence of baricitinib. Ectopic expression of constitutively active STAT3 completely abrogated the synergistic effects of baricitinib in combination with docetaxel (Figure 5A,B). In contrast, no such effects were observed in cells transfected with the constitutively active myristoylated AKT (myrAKT) (Figure 5C,D). Together, these results suggest that the combinations of JAK1 inhibitors with docetaxel possess synergistic effects in PCa cells via inactivation of STAT3.

## 4 DISCUSSION

Recent clinical studies have demonstrated the clear advantage of docetaxel over other chemotherapeutic agents in the treatment of prostate cancer.^6^, ^61^, ^62^, ^63^, ^64^ However, dose‐limiting adverse effects, including neutropenia, myelosuppression and neurotoxicity,^65^ are the main reasons that docetaxel treatment is halted at a lower cumulative dose, despite evidence showing that patients receiving 10 or more cycles of docetaxel had the greatest median overall survival compared to patients receiving 8 to 10 or 5‐7 cycles.^6^

Previous studies have attempted to improve docetaxel's efficacy and to reduce its toxicity by combining with various therapeutic agents with distinct mechanisms of action, including tyrosine kinase inhibitors (eg dasatinib), endothelin receptor antagonist (eg atrasentan and zibotentan), angiogenic inhibitors (eg bevacizumab and aflibercept), BCL2 inhibitors and immunologic agents (eg GVAX and lenalidomide).^7^, ^9^, ^10^, ^11^, ^12^, ^13^, ^66^, ^67^, ^68^ However, no drug has yet demonstrated improved overall survival when added to docetaxel in a phase III trial, and in some cases, the addition proved detrimental to outcomes.

We hypothesized that synthetic lethality could be achieved by combining docetaxel with selective kinase inhibitors since such agents can be amenable to drug development in the event of specific vulnerabilities being identified. Thus, we designed a high‐throughput kinome‐wide loss‐of‐function shRNA screening combined with pathway analysis to identify combinatorial synthetic lethal and resistant interactions, specifically druggable targets that could enhance docetaxel sensitivity in PCa cells. We observed that cells deficient in 15 kinases become hypersensitive to docetaxel and identified JAK1 and IGF1R as potential druggable PCa targets. We have also identified experimental inhibitors and FDA‐approved drugs targeting JAK1, providing opportunities for drug repurposing.

Although the oncogenic functions of IGF1R have been previously demonstrated in PCa ^30^, ^31^, ^69^ and inhibition of IGF1R reduced PCa cell growth in in vitro and in vivo models,^33^, ^34^, ^35^, ^36^ targeting IGF1R with cixutumumab failed to improve the survival outcome of metastatic PCa.^53^ Similarly, addition of figitumumab to the standard regimen of docetaxel/prednisone seemed to have detrimental impact on clinical outcomes due to increased treatment‐related toxicity (eg hyperglycaemia, diarrhoea and asthenia), highlighting the challenges of improving the activity of docetaxel by targeting IGF1R.^54^

Recently, JAKs have emerged as an attractive therapeutic target in various human cancers as JAKs are key player in signalling networks driving cancer cell proliferation, survival, invasiveness and metastasis and suppressing the antitumor immune response.^70^, ^71^ JAKs comprise of four family members (JAK1, JAK2, JAK3 and TYK2) and interact with a variety of cytokine and growth factor receptors (eg GP130, IFNγR, IFNαR, IL‐10/20R, and CXCR4).^70^, ^71^ Upon ligand binding to their cognate receptors, JAKs are activated via reciprocal trans‐phosphorylation and mediate intracellular signalling cascades through phosphorylation of STATs (STAT1, STAT2, STAT3, STAT4, STAT5A, STAT5B and STAT6).^71^ In turn, tyrosine‐phosphorylated STATs form homo‐ or heterodimers translocate into the nucleus and elicit specific transcriptional programme.^71^

Inhibition of JAKs has been shown to reduce tumour growth in various in vitro and in vivo models, including brain, breast, colorectal, gastric, head‐and‐neck, liver, lung, pancreatic, ovarian and prostate cancers.^37^, ^38^, ^72^, ^73^, ^74^, ^75^, ^76^, ^77^ Interestingly, combined inhibition of JAK1/2, STAT3 and PD‑L1 was shown to suppress the immune evasion of CRPC to NK cells in hypoxia.^57^ Suppression of JAK1/2 by AZD1480 also inhibited progression of CRPC ^37^ and reduced IL6‐induced metastases,^38^ suggesting that JAK signalling is activated in prostate cancer.

Indeed, our results demonstrated that JAK1/2 inhibitors, ruxolitinib and baricitinib, selectively inhibited the AR‐negative DU145 and PC3 cell proliferation as compared to the AR‐positive LNCaP or RWPE‐1 normal prostate epithelial cells. Notably, ruxolitinib and baricitinib also synergized docetaxel sensitivity in the AR‐negative DU145 and PC3 cells, while only additive effects were observed in the AR‐positive LNCaP cells. In contrast, the JAK2‐specific inhibitor, fedratinib, elicited mainly additive and antagonistic effects with docetaxel in all the PCa cell lines being tested. Of note, ruxolitinib, baricitinib and fedratinib are FDA‐approved drugs for treatment of myelofibrosis, active rheumatoid arthritis and myeloproliferative neoplasm‐associated myelofibrosis, respectively, suggesting a potential drug repurposing of these agents for the treatment of prostate cancers.^78^, ^79^, ^80^

Several studies have shown that IL6/JAK/STAT3 signalling plays a central role in regulating docetaxel sensitivity.^81^, ^82^, ^83^ Indeed, both the AR‐negative DU145 and PC3 (but not in LNCaP cells) have been shown to have elevated IL6 production leading to activated JAK‐STAT signalling and constitutive NFκB activity, rendering these cells resistant to docetaxel.^81^, ^82^, ^84^, ^85^, ^86^, ^87^, ^88^ These observations may explain the lack of synergism between JAK1/2 inhibitors with docetaxel in LNCaP cells as compared to the JAK‐STAT ‘addicted’ DU145 and PC3 cells. Indeed, inhibition of JAK1 by ruxolitinib and baricitinib reduced STAT3 phosphorylation, while ectopic expression of a constitutively active STAT3C significantly abrogated the synergistic effects of the baricitinib/docetaxel combination in both DU145 and PC3 cells. In contrast, no such effect was observed in cells overexpressing a myristoylated AKT, suggesting that the synergistic effects of docetaxel and JAK1 inhibitors mediated mainly through inhibition of STAT3 signalling.

## 5 CONCLUSIONS

In conclusion, we showed that inhibition of JAK1 synergizes with docetaxel sensitivity in AR‐negative PCa cells via inhibition of STAT3 signalling. Overall, our findings suggest that combination therapy with JAK1/2 inhibitors and docetaxel may be a useful approach for treating patients with advanced PCa and warrant further investigation in the future in vivo models.

## CONFLICTS OF INTEREST

The author(s) declare no competing interests. Where authors are identified as personnel of the International Agency for Research on Cancer/World Health Organization, the authors alone are responsible for the views expressed in this article and they do not necessarily represent the decisions, policy or views of the International Agency for Research on Cancer/World Health Organization.

## AUTHOR CONTRIBUTIONS

**Geetha Nalairndran:** Investigation (lead); Methodology (lead); Writing‐original draft (equal); Writing‐review & editing (equal). **Ivy Chung:** Conceptualization (equal); Funding acquisition (equal); Project administration (equal); Supervision (equal); Writing‐review & editing (equal). **Azad Hassan Abdul Razack:** Conceptualization (equal); Funding acquisition (equal); Project administration (equal); Supervision (equal). **Felicia Fei‐Lei Chung:** Investigation (equal); Methodology (equal); Writing‐review & editing (equal). **Ling‐Wei Hii:** Investigation (equal); Methodology (equal); Writing‐review & editing (equal). **Wei‐Meng Lim:** Investigation (equal); Methodology (equal); Writing‐review & editing (equal). **Chin King Looi:** Investigation (equal); Methodology (equal). **Chun‐Wai Mai:** Investigation (equal); Methodology (equal); Writing‐review & editing (equal). **Chee‐Onn Leong:** Conceptualization (equal); Funding acquisition (equal); Project administration (equal); Supervision (equal); Writing‐original draft (equal); Writing‐review & editing (equal).

## Supporting information

Table S1‐S3

## Data Availability

The data that support the findings of this study are available in the supplementary material of this article.
