# Supplementary material for: Inhibition of Janus Kinase 1 synergizes docetaxel sensitivity in prostate cancer cells
Source: J Cell Mol Med. 2021 Jul 28;25(17):8187–200. doi: 10.1111/jcmm.16684 (PMC8419172; doi:10.1111/jcmm.16684)
Supplement: Supplementary file 1 — Table S1‐S3 [file JCMM-25-8187-s001.docx]

**SUPPLMENTAL TABLES**

**Supplemental Table 1**. Range of Combination Index (CI)

| **CI Range** | **Description** |
| --- | --- |
| < 0.1 | Very strong synergism |
| 0.1 - 0.3 | Strong synergism |
| 0.3 - 0.7 | Synergism |
| 0.7 - 0.85 | Moderate synergism |
| 0.85 - 0.90 | Slight synergism |
| 0.90 - 1.10 | Nearly additive |
| 1.10 - 1.20 | Slight antagonism |
| 1.20 - 1.45 | Moderate antagonism |
| 1.45 - 3.3 | Antagonism |
| 3.3 - 10 | Strong antagonism |
| >10 | Very strong antagonism |

**Supplemental Table 2.** shRNAs target sequences

| **shRNAs** | **Target Sequence** |
| --- | --- |
| JAK1-si1 | 5’-AATCAGTAACATGGAAGTCTC-3’ |
| JAK1-si2 | 5’-TTTATCCTCCAAGTAGCTCAG-3’ |

**Supplemental Table 3:** Half maximal inhibitory concentration (IC_50_) values of docetaxel and JAK1/2 inhibitors against PCa and normal prostate epithelial cells.

| **Cell lines** | **Docetaxel (nM)** | **Ruxolitinib (µM)** | **Baricitinib (µM)** | **Fedratinib (µM)** |
| --- | --- | --- | --- | --- |
| RWPE-1 | < 0.16 | >100 | >100 | >100 |
| LNCaP | 0.88 ± 0.12 | >100 | >100 | >100 |
| DU145 | > 10 | 13.80 ± 2.67 | 16.52 ± 1.40 | 8.88 ± 1.11 |
| PC3 | > 10 | 47.52 ± 1.56 | 52.56 ± 1.64 | 28.99 ± 1.73 |
